# Outbreak of *Pseudomonas aeruginosa* High-Risk Clone ST309 Serotype O11 Featuring *bla*_PER-1_ and *qnrVC6*

**DOI:** 10.3390/antibiotics13020159

**Published:** 2024-02-06

**Authors:** Romina Papa-Ezdra, Matilde Outeda, Nicolás F. Cordeiro, Lucía Araújo, Pilar Gadea, Virginia Garcia-Fulgueiras, Verónica Seija, Inés Bado, Rafael Vignoli

**Affiliations:** 1 Departamento de Bacteriología y Virología, Instituto de Higiene, Facultad de Medicina, Universidad de la República, Av. Alfredo Navarro 3051, CP 11600 Montevideo, Uruguay; rpapa@higiene.edu.uy (R.P.-E.); ncordeiro@higiene.edu.uy (N.F.C.); laraujo@higiene.edu.uy (L.A.); virginiagarcia@higiene.edu.uy (V.G.-F.); 2 Departamento de Laboratorio Clínico, Área Microbiología, Hospital de Clínicas, Facultad de Medicina, Universidad de la República, Av. Italia s/n, CP 11600 Montevideo, Uruguay; matildeouteda@gmail.com (M.O.); pilargadea2009@gmail.com (P.G.); vseija1@gmail.com (V.S.)

**Keywords:** *Pseudomonas aeruginosa*, ESBL, *bla*_PER-1_, transposon, ST309

## Abstract

*Pseudomonas aeruginosa* is a leading cause of hospital-acquired infections worldwide. Biofilm production, antibiotic resistance, and a wide range of virulence factors contribute to their persistence in nosocomial environments. We describe an outbreak caused by a multidrug-resistant *P. aeruginosa* strain in an ICU. Antibiotic susceptibility was determined and *bla*_PER-1_ and *qnrVC* were amplified via PCR. Clonality was determined using PFGE and biofilm formation was studied with a static model. A combination of antibiotics was assessed on both planktonic cells and biofilms. WGS was performed on five isolates. All isolates were clonally related, resistant to ceftazidime, cefepime, amikacin, and ceftolozane-tazobactam, and harbored *bla*_PER-1_; 11/19 possessed *qnrVC*. Meropenem and ciprofloxacin reduced the biofilm biomass; however, the response to antibiotic combinations with rifampicin was different between planktonic cells and biofilms. WGS revealed that the isolates belonged to ST309 and serotype O11. *bla*_PER-1_ and *qnrVC6* were associated with a tandem of IS*CR1* as part of a complex class one integron, with *aac(6′)-Il* and *ltrA* as gene cassettes. The structure was associated upstream and downstream with Tn*4662* and flanked by direct repeats, suggesting its horizontal mobilization capability as a composite transposon. ST309 is considered an emerging high-risk clone that should be monitored in the Americas.

## 1. Introduction

Antimicrobial resistance (AMR) is one of the main health challenges of the 21st century, which threatens to claim millions of lives annually and causes significant health costs in terms of gross domestic product (GDP), according to projections for the next 30 years [1]. Recent studies estimate that 4.5 million deaths were associated with and 1.27 million were attributable to bacterial AMR in 2019 [2].

*Pseudomonas aeruginosa* ranks among the six leading pathogens contributing to AMR-associated deaths and is one of the five pathogens most related with mortality and years of life lost, independently of AMR [2,3]. Although this Gram-negative rod is considered an opportunistic pathogen, *P. aeruginosa* is one of the main agents of hospital-acquired infections worldwide; moreover, it plays a key role in infections among immunocompromised patients, in patients with cystic fibrosis and burns, among others [4,5]. Lower respiratory tract and bloodstream infections followed by peritoneal and intra-abdominal infections and, to a lesser extent, urinary tract infections and infections of the skin and subcutaneous systems are the main syndromes associated with mortality caused by this microorganism [3].

The high associated mortality of *P. aeruginosa* can be attributed to several factors, including the frequent occurrence of diverse antibiotic resistance mechanisms, its ability to produce biofilm [6], its association with certain virulence factors [7], and its ability to persist in hospital and natural environments [8]. This is particularly noteworthy in high-risk clones (HRCs), a term often used to refer to multidrug-resistant or extensively drug-resistant *P. aeruginosa* clones with wide distribution, usually associated with epidemic outbreaks, and exemplified by ST235, ST111, ST233, ST244, ST357, ST308, ST175, ST277, ST654, and ST298 [7]. HRCs are often linked with the production of extended-spectrum β-lactamases (ESBLs) or carbapenemases, along with certain serotypes (especially O11 and O6) and potent exotoxins associated with type three secretion systems (T3SSs), such as ExoU or ExoS [7]. Recently, several authors have proposed the inclusion of the sequence type ST309 among the HRCs because it meets multiple characteristics described above and because of its wide dissemination [8,9,10].

Moreover, many isolates belonging to these HRCs are considered within the category of *P. aeruginosa* with Difficult-to-Treat Resistance (DTR-*P. aeruginosa*), a term recently proposed to denote isolates that exhibit non-susceptibility to all of the following antibiotics: piperacillin-tazobactam, ceftazidime, cefepime, aztreonam, meropenem, imipenem, ciprofloxacin, and levofloxacin [11].

As most of these antibiotics belong to the β-lactam family, the main mechanism of resistance, alongside low outer membrane permeability, lies in the production of specific β-lactamases such as ESBLs and carbapenemases. Among the ESBLs and carbapenemases reported in this microorganism, those acquired horizontally, such as CTX-M-2, PER-1 or variants of VIM, IMP, and GES, are noteworthy for their frequency and association with HRCs [7].

Class one integrons play a key role in disseminating carbapenemase- and ESBL-coding genes in *P. aeruginosa*, contributing to the development of multidrug resistance, by co-harboring resistance genes to other antibiotic groups, including aminoglycosides and fluoroquinolones [12]. These elements consist of two conserved segments, 5′-CS and 3′-CS, flanking a variable region where resistance genes are incorporated as gene cassettes. The 5′-CS contains the class one integrase-coding gene (*intI1*), promoters for the expression of *intI1* (Pint) and the gene cassettes (Pc), and the integrase recognition site (*attI1*) that is recognized by the integrase to mediate site-specific recombination, along with the cassette recognition site (*attC*), to incorporate and excise genes to the structure. In clinically relevant class one integrons, also known as ‘sul1-type’ integrons, the 3′-CS region typically consists of a truncated version of the quaternary ammonium compounds resistance gene *qacE1* (Δ*qacE1*) and the sulfonamide resistance gene *sul1* [13,14].

Concerning the structures from which class one integrons are derived, In*4*-like integrons are especially frequent in *P. aeruginosa* [12]. These structures include IS*6100* at the end of the 3′-conserved segment (3′-CS), which may be truncated or absent due to this insertion sequence. Moreover, additional resistance genes can appear through their association with the IS*CR1* element, followed by a partial duplication of the 3′-CS, constituting the so-called complex class one integrons [15]. On the other hand, although most class one integrons have lost their transposition functions, they can be mobilized when associated with Tn*3*-like transposons [12,14].

Previously, *P. aeruginosa* clinical isolates carrying the carbapenemase-coding gene *bla*_VIM-2_ in class one integrons were reported from different settings in Uruguay [16]. Subsequently, its co-occurrence with *bla*_PER-1_ was described, associated with novel resistance regions and transposition units [17]. In this study, we describe an outbreak caused by multidrug-resistant *P. aeruginosa* in an Intensive Care Unit (ICU), and characterize the molecular and microbiological features of the clone involved. 

## 2. Results

### 2.1. Isolates

A total of 43 *P. aeruginosa* isolates were obtained from nineteen infected and four colonized patients (based on the clinical criteria established by the infectious diseases team), admitted to the Intensive Care Unit (ICU) of the Hospital de Clínicas of Montevideo between August 2021 and July 2022. Antibiotic susceptibility testing and pulsed-field gel electrophoresis (PFGE) were conducted on the first isolate obtained from each patient. As the four isolates from the colonized patients exhibited the same antibiotic susceptibility patterns and pulse types (as detailed below), they were excluded from subsequent microbiological studies. The 19 studied isolates were obtained from respiratory secretions (n = 13), blood culture (n = 3), bronchoalveolar lavage (n = 1), urine (n = 1), and a surgical wound (n = 1) (Table 1). 

### 2.2. Pulsed-Field gel Electrophoresis

**Figure 1:**
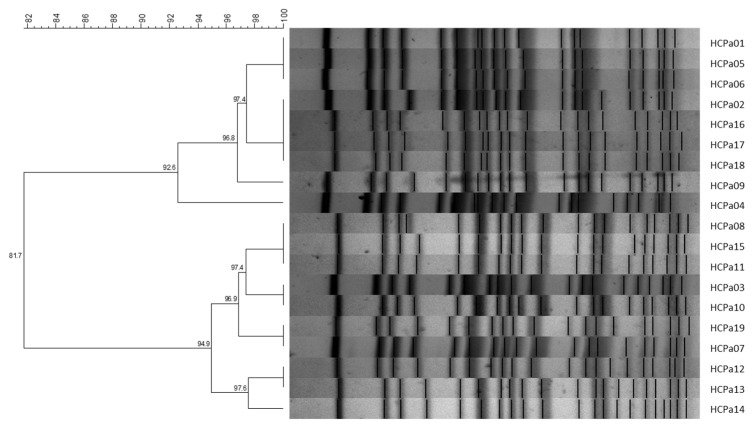
UPGMA dendrogram with Dice similarity coefficient; 1% opti., 2% tol. Similarity coefficients (%) are indicated in each node. Generated with BioNumerics v6.6 software.

All 19 isolates were clonally related, exhibiting similarity coefficients >80%. Grouping analysis further identified the presence of two sub-clusters comprising nine and ten isolates, respectively, with similarity coefficients >90% (Figure 1). Also, the strains corresponding to colonization exhibited a compatible pattern with the clinical isolates, displaying similarity coefficients >80%. 

### 2.3. Susceptibility Profiles and Mechanisms of Antibiotic Resistance

All isolates were resistant to ceftazidime, cefepime, and amikacin, while three were also resistant to ciprofloxacin. Additionally, four strains were resistant to imipenem and one of them was resistant to both imipenem and meropenem. Five strains were not susceptible to piperacillin-tazobactam and four to gentamicin (Table 1).

Additionally, isolates from colonized patients exhibited a similar susceptibility profile, characterized by resistance to ceftazidime, cefepime, and amikacin. They were susceptible to imipenem, meropenem, gentamicin, and ciprofloxacin, and either susceptible or intermediate to piperacillin-tazobactam. 

Testing newer antibiotics, not widely available in our country, revealed resistance to ceftolozane-tazobactam and susceptibility to cefiderocol among all isolates. Regarding ceftazidime-avibactam, five isolates were susceptible (MIC = 8 mg/L) and the remaining fourteen were resistant (Table 1).

The double-disk synergy test between amoxicillin-clavulanic acid and ceftazidime resulted positive for all 19 isolates, and the presence of the ESBL-coding gene *bla*_PER-1_ was identified using PCR in all of them. Additionally, the quinolone resistance determinant *qnrVC6* was detected in 11/19 isolates (Table 1). Isolates from the colonized patients also harbored the *bla*_PER-1_ gene.

### 2.4. Effect of Antibiotics Combined with Rifampicin

The combined effects of either ciprofloxacin, meropenem, gentamicin, and amikacin with rifampicin was evaluated using the checkerboard assay on two strains, HCPa01 and HCPa12. A synergistic effect (FICI ≤ 0.5) was observed for all the combinations except for ciprofloxacin plus rifampicin. Meropenem 2 mg/L and rifampicin 4 mg/L resulted synergistic for strain HCPa01 (FICI = 0.5), meanwhile meropenem 0.06 and 0.5 mg/L were synergistic with rifampicin 2 and 4 mg/L (FICI = 0.375 and 0.28), respectively, for strain HCPa12. For both strains, gentamicin and rifampicin resulted synergistic at 0.5 and 4 mg/L (FICI = 0.375), meanwhile amikacin 8 mg/L exhibited synergy with rifampicin 4 mg/L (FICI = 0.375) in both strains. Furthermore, HCPa12 demonstrated synergy with amikacin 16 mg/L and rifampicin 0.5 mg/L (FICI = 0.28) (Table 2).

### 2.5. Biofilm Characterization

HCPa01 and HCPa12 were categorized as strong biofilm producers at 24 h, exhibiting OD590 = 0.789 ± 0.11 and 0.674 ± 0.096, respectively (ODc = 0.126 ± 0.022). 

**Figure 2:**
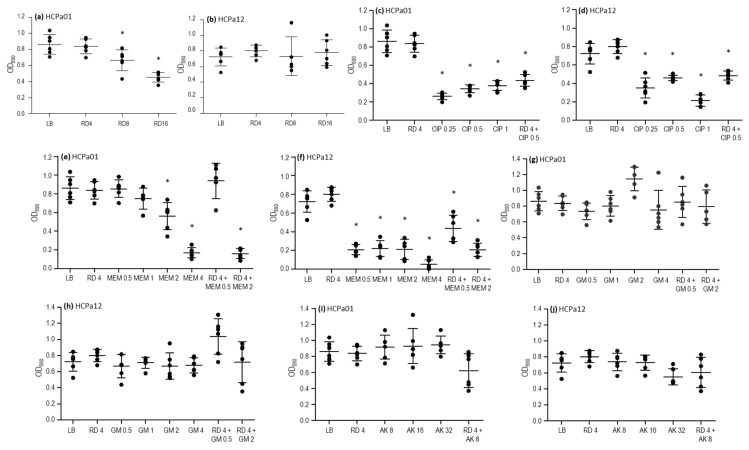
Activity of antibiotics alone and in combinations over 24 h on the mature biofilm of strains HCPa01 and HCPa12. Biofilm biomass is expressed as optical density measured at 590 nm (OD_590_), antibiotic concentrations in mg/L (*x* axis). (**a**,**b**) Rifampicin (RD) alone; (**c**,**d**) ciprofloxacin (CIP) alone and combined with RD; (**e**,**f**) meropenem (MEM) alone and combined with RD; (**g**,**h**) gentamicin (GM) alone and combined with RD; (**i**,**j**) mikacin (AK) alone and combined with RD. The control without antibiotics is indicated as LB. Asterisks (*) indicate a significative decrease (*p* < 0.05) in biofilm biomass in comparison with the control without antibiotics.

To further characterize the strains, the activity of ciprofloxacin, meropenem, gentamicin, amikacin, and rifampicin was assessed against 24 h mature biofilms. Additionally, combinations that resulted synergistic in the checkerboard analysis (meropenem, gentamicin, and amikacin plus rifampicin) were evaluated too, along with a combination of rifampicin and ciprofloxacin. Rifampicin at concentrations of 8 and 16 mg/L led to a reduction in the biofilm biomass in HCPa01, compared with the control without antibiotics (*p* < 0.05), although this effect was not observed in HCPa12 (Figure 2a,b). Ciprofloxacin 0.25, 0.5, and 1 mg/L resulted in a reduction in biofilm biomass (*p* < 0.05) in both strains, as well as the combination of ciprofloxacin 0.5 mg/L and rifampicin 4 mg/L, although this effect was not statistically significant compared to ciprofloxacin alone (Figure 2c,d). 

Regarding meropenem, the biofilm biomass of strain HCPa01 decreased with 2 and 4 mg/L, while in HCPa12 0.5, 1, 2, and 4 mg/L produced a decrease in biomass compared to the control without antibiotics (*p* < 0.05). The combination of meropenem 2 mg/L plus rifampicin 4 mg/L also resulted in a reduction (*p* < 0.05) in biofilm biomass compared to the control without antibiotics and with rifampicin alone in both strains, as well as with meropenem alone in the case of HCPa01. The combination of meropenem 0.5 mg/L with rifampicin 4 mg/L showed a reduction in the biomass of the strain HCPa12 compared to the control without antibiotics and rifampicin alone, but it exhibited a biomass increase compared to meropenem 0.5 mg/L alone, although not statistically significant; meanwhile, such combination showed no effect against the biofilm of HCPa01 (Figure 2e,f).

Gentamicin 0.5, 1, 2, and 4 mg/L and amikacin 8, 16, and 32 mg/L showed no effect against the biofilms of both strains. Neither the combinations of gentamicin 0.5 or 2 mg/L, or amikacin 8 mg/L with rifampicin 4 mg/L evidenced a biofilm reduction. Conversely, an increase in the biomass of HCPa12 was observed when treated with 4 mg/L rifampicin and 0.5 mg/L gentamicin (Figure 2g–j).

### 2.6. Genetic Features

Whole genome sequencing (WGS) was performed on five isolates (HCPa01, HCPa02, HCPa10, HCPa12, and HCPa16). Sequence analyses revealed they all belonged to the sequence type ST309 and serotype O11. 

Analysis using the Virulence Factor Database (VFDB) identified more than 200 virulence-related determinants in the five strains. Among the most relevant genes were those associated with type III secretion system effectors and regulators, including the toxin *exoU*, and adherence factors related with type IV pili and flagella biosynthesis, as well as their regulation and components. Additionally, genes were identified for alginate biosynthesis and regulation, elastase, rhamnolipid, and pyocyanin biosynthesis. Other genes related to the type IV and VI secretion systems, pyochelin, and pyoverdine synthesis and regulation were also found. 

Regarding the antibiotic resistance determinants, AMRFinder revealed the presence of ten resistance determinants including *aac(6′)-Il* (*aacA7*), *aph(3′)-IIb* (aminoglycoside resistance), *bla*_OXA-1035_ (OXA-50 family), *bla*_PDC-19a_, *bla*_PER-1_ (β-lactam resistance), *catB7* (phenicol resistance), *fosA* (fosfomycin resistance), *qacEΔ1* (quaternary ammonium resistance), and *sul1* (sulfonamide resistance) in all five strains studied, meanwhile the quinolone resistance determinant *qnrVC6* was detected in all strains except HCPa12. Finally, four isolates presented the *crpP1* locus variant, while HCPa01 had the *crpP* variant.

### 2.7. qnrVC6 and bla_PER-1_ Genetic Environment

**Figure 3:**
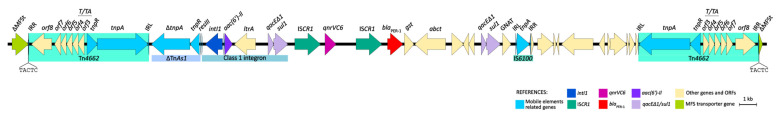
Genetic environment of *qnrVC6* and *bla*_PER-1_ in *P. aeruginosa* HCPa10, featuring a composite transposon (Tn*7723*) delimited by two copies of Tn4662 and flanked by direct repeats (5′-TACTC). Genes and ORFs are represented by arrows and colored according to their function, as indicated in the reference. Linear map generated in EasyFig v2.1.

The genetic environments of both *qnrVC6* and *bla*_PER-1_ were studied in detail in the strain HCPa10, and consist of a 43.3 kb structure. Both genes are embedded into a complex class one integron comprising the class one integron *intI1*–*aac(6′)-Il*–*ltrA*–*qacEΔ1*-*sul1*, followed by a tandem structure consisting of IS*CR1*–*qnrVC6*–IS*CR1*–*bla*_PER-1_, concluding in a second copy of *qacEΔ1*-*sul1*. Upstream of this structure there is a reverse-oriented transposon belonging to the Tn*3* family, named Tn*4662*, which comprises transposase and resolvase genes (*tnpA* and *tnpR*, respectively), the resolvase system formed by three *res* sites (I, II and III), a toxin/antitoxin gene pair (*relE* and *relB*), and four additional ORFs, all flanked by the inverted repeats IRL and IRR. Adjacent to the Tn*4662* and immediately upstream of *intI1*, there is a fragment of Tn*As1* comprising the truncated transposase gene (Δ*tnpA*), the resolvase gene (*tnpR*), and the *res* site RIII. Finally, downstream of the complex class one integron there is the insertion sequence IS*6100*, with a second copy of Tn4662 positioned 6.4 kb apart and directly oriented. Upstream of the first copy of Tn*4662* and downstream the second, there are 5 bp direct repeats (DRs) (5′-TACTC), flanking the composite transposon designated as *Tn*7723. Moreover, upstream of the first DR there are 979 bp of an MFS transporter-coding gene, and downstream the second DR there are the remaining 228 bp of the same gene (Figure 3).

**Figure 4:**
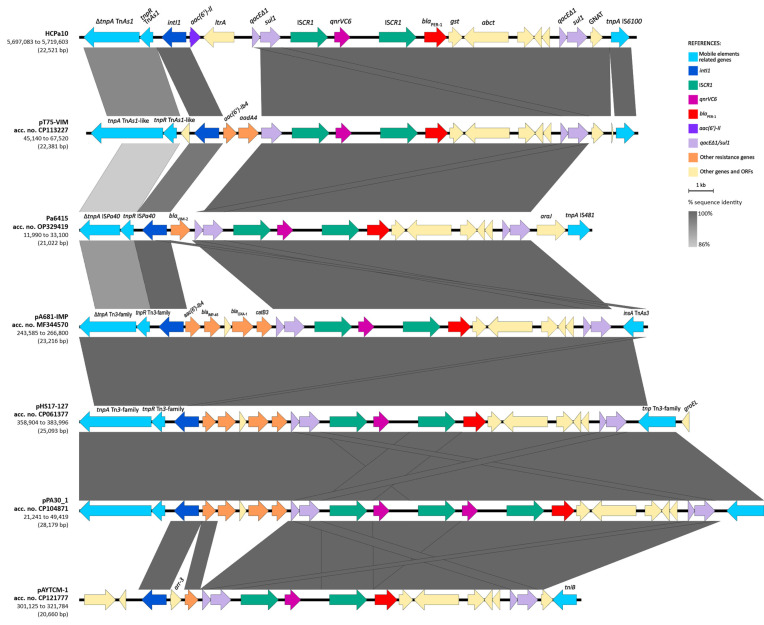
Sequence comparison (BLAST) of the resistance region harboring *qnrVC6* and *bla*_PER-1_ with other genetic platforms from GenBank. Genes and ORFs are represented by arrows and colored according to their function and homologous segments are represented in shades of gray according to sequence identity, both as indicated in the reference. Linear map generated in EasyFig v2.1.

The BLAST analysis of the structure in the GenBank database revealed that the overall structure of the genetic environment of *qnrVC6*–*bla*_PER-1_ is similar to others previously described in three plasmids and one chromosome of *Pseudomonas aeruginosa*, one *Pseudomonadaceae* plasmid, and one *Acinetobacter johnsonii* plasmid (Figure 4). The platform is mostly composed of a class one integron, with different gene cassettes among the isolates, followed by the module IS*CR1*–*qnrVC6*–IS*CR1*–*bla*_PER-1_–*gst*–*abct–qacEΔ1*-*sul1*, and generally associated upstream with Tn*3*-family derived resolvase and transposase genes (complete or partial). The integron identified in HCPa10 represents a new class one integron (5′-CS–*aac(6′)-Il*–*ltrA*–3′-CS), and is also novel to the platform, since the aforementioned isolates were associated with different gene cassettes, including *aac(6′)-Ib4–aadA4* (in CP113227), *bla*_VIM-2_ (in OP329419), *aac(6′)-Ib4–bla*_IMP-45_–*bla*_OXA-1_–*catB3* (in MF344570, CP061377 and CP104871), or *arr-3* (in CP121777). 

## 3. Discussion

*P. aeruginosa* is listed among the leading bacterial pathogens responsible for infection-related deaths in recent years, and is a significant contributor to the burden of AMR [2,3]. Lower respiratory infections and bloodstream infections stand out as the main causes of death attributed to this pathogen [3]. This microorganism is particularly frequent as a cause of nosocomial infections in ICUs [18]. 

In our study, *P. aeruginosa* was predominantly isolated from respiratory secretions, followed by blood samples from patients admitted to an ICU. All isolates were resistant to ceftazidime, cefepime, and amikacin. It is noteworthy that both ceftazidime and cefepime are among the most used antibiotics in this setting, while meropenem is rarely administered. Moreover, newer antibiotics such as ceftolozane/tazobactam and cefiderocol are not yet available in Uruguay, or their use is restricted to particular cases, as is the case of ceftazidime/avibactam. It has been suggested that the use of older antipseudomonal β-lactams such as ceftazidime, cefepime, and piperacillin/tazobactam may exert selective pressure, contributing to the emergence of resistance to newer agents like ceftolozane/tazobactam [19]. 

The high-level resistance to both ceftazidime and cefepime observed in all isolates can be attributed to the presence of the ESBL-coding gene *bla*_PER-1_. Moreover, the expression of PER-1 may also explain the resistance to ceftolozane/tazobactam and ceftazidime/avibactam [20,21]. Interestingly, although *bla*_PER-1_ is most frequently detected in *P. aeruginosa* isolates from Europe and the Middle East, to our knowledge, it has only been reported from Uruguay and Chile in the Americas [17,22,23]. 

On the other hand, although the quinolone resistance gene *qnrVC6* was found in half of the isolates, most of them were susceptible to ciprofloxacin, which may be expected under the assumption that generally, *qnr* genes confer low level quinolone resistance [24]. Also, the isolates analyzed via WGS harbored either *crpP* or *crpP1* locus variants, However, neither of these have been associated with fluoroquinolone resistance [25]. The resistance to amikacin can be attributed to the presence of the aminoglycoside N-acetyltransferase (6′) type 1 gene *aac(6′)-Il* (*aacA7*) [26]. Discrepancies in antibiotic susceptibility observed among strains isolated from different patients may be explained by the development of adaptative resistance, mainly triggered by antibiotic selective pressure, and differences in gene regulation and expression [12].

In this study, we describe an outbreak of *P. aeruginosa* in an ICU involving 23 patients. This microorganism is well documented as a cause of outbreaks within such healthcare settings, mainly due to its capacity to colonize and survive in different surfaces [10]. The biofilm formation capacity is a key strategy for environmental colonization, which is also associated with higher antibiotic resistance and persistent infections, as well as to the clonal success of high-risk clones [27]. 

Both strains selected for biofilm formation analysis demonstrated strong biofilm-producing capabilities and exhibited variations in antibiotic susceptibility when comparing biofilm and planktonic growth stages. Notably, both ciprofloxacin and meropenem demonstrated the ability to reduce the biofilm biomass when assessed individually; meanwhile, the combination with rifampicin did not yield additional effects. Aminoglycosides, when evaluated individually or combined with rifampicin, did not exhibit a significant impact on biofilm biomass. Conversely, in planktonic cells, the combination of either meropenem, gentamicin, or amikacin showed a synergistic effect. Although combination therapy with rifampicin has proven effective in treating staphylococcal biofilms [28], and even in vitro against carbapenemase-producing *Escherichia coli* and *Klebsiella pneumoniae* [29], limited evidence exists regarding its efficacy against *P. aeruginosa* biofilms. Previous studies have shown positive in vitro outcomes when combining carbapenems and rifampicin at sub-MIC concentrations [29], and a rifampicin-driven potentiation of aminoglycoside activity [30]. However, in both cases, the assays were conducted with planktonic *P. aeruginosa*. Further studies are needed to further understand the role of rifampicin against *P. aeruginosa* in both planktonic and biofilm forms. 

In this work, both *bla*_PER-1_ and *qnrVC6* were located in a genetic platform consisting of a tandem with two copies of the IS*CR1* element. This arrangement was associated upstream to a class one integron harboring *aac(6′)-Il* and *ltrA* as gene cassettes, and downstream with a second copy of *qacEΔ1*/*sul1*, constituting a complex class one integron. Similar configurations were identified in the database, differing only in the gene cassettes carried by the class one integrons. These variations included either aminoglycoside, chloramphenicol, or β-lactam resistance genes such as *aac(6′)-Ib4*, *aadA4*, *catB3*, *bla*_OXA-1_, *bla*_IMP-45_, or *bla*_VIM-2_, the latter reported recently by our group in a clinical *P. aeruginosa* obtained from the same setting [17]. Upstream of the class one integron, most platforms were associated with different Tn*3*-family elements, typically featuring a resolvase gene and a complete or truncated transposase gene. Meanwhile, the elements found downstream comprised diverse genes derived from various transposon families such as IS*6* (IS*6100* in our case), Tn*3,* or IS*481*. Notably, the presence of IS*6100* is characteristic of In*4*-like class one integrons, which usually lack part of all of the 3′-CS region, including the IRt [12], this latter being absent in the platform described here. 

Interestingly, the whole region is bounded by two oppositely oriented transposons known as Tn*4662*. These transposons are flanked upstream and downstream by 5 pb direct repeats, interrupting an MFS transporter gene. This suggests that the entire structure might have undergone mobilization as a composite transposon, denoted here as Tn*7723.* This mobilization pattern as has been previously demonstrated for other Tn*3*-family derived structures [31]. 

As suggested by data obtained from the WGS of five representative strains, the outbreak described here was caused by *P. aeruginosa* belonging to sequence type ST309 and serotype O11. ST309 has recently been proposed as a high-risk clone given its wide distribution among different continents including Asia, Europe, Oceania, and the Americas [8,32]. This lineage was initially identified in low proportions as a minor clone in Greece and Korea, with the latter being associated with *bla*_VIM-2_; subsequent reports have documented clonal spread. These include a massive and persistent colonization of a dental care unit waterline in a University Hospital in France [33], isolates from children with bacteremia in Mexico [10], extensively drug-resistant clinical isolates from the United States [9], the Philippines [34], and Brazil [8], and intestinal colonization and environmental samples from a long-term care facility in France [32]. ST309 clones are reported to carry several acquired resistance genes in class one integrons, including ESBLs (*bla*_GES-19, -20, -26_), carbapenemases (*bla*_VIM-2_ and *bla*_IMP-15_), aminoglycoside-modifying enzymes (*aadA1*, *aacA4*, *aac(6′)-Il* and *aac(6′)-33*), and fluoroquinolone resistance genes (*qnrVC1*), among others [8]. In our case, the ESBL detected among all isolates was *bla*_PER-1_, accompanied by *qnrVC6* within a complex class one integron, comprising novel components to the resistome of the ST309 lineage.

Regarding the serotype O11, it stands out as one of the most frequent serotypes among *P. aeruginosa* isolates worldwide, alongside O1 and O6 [35]. It has been frequently reported among high-risk clones such as ST235, ST357, ST308, and ST298 [7]. Serotype O11 was related with high prevalence among critically ill patients [35], worse clinical outcomes, extended hospital stays, and more virulent phenotypes [36]. As for virulence, the exotoxin ExoU, secreted by the type III secretion system (T3SS), is a key virulence factor of *P. aeruginosa* pathogenicity [18] and has been frequently reported in O11 isolates [35,36]. Both the gene *exoU* and T3SS-coding genes were detected in our isolates. 

The clone here described exhibits concerning characteristics, including antibiotic resistance genes associated with transferable elements, several virulence factors, a resistance to new antibiotics, and the ability to form biofilms. This outbreak persisted for at least one year in the ICU, suggesting the presence of an environmental source as documented in previous studies, where biofilms could play an essential role [27,32,33]. Additionally, an enhanced ability to develop biofilms has been proposed as an underlying factor behind the success of high-risk clones [7]. 

In summary, we describe a large hospital outbreak caused by a successful high-risk clone of *P. aeruginosa* ST309. This clone carries resistance genes to broad-spectrum cephalosporins, amikacin, and new β-lactam/β-lactamase inhibitor combinations such as ceftolozane-tazobactam and ceftazidime-avibactam. This study highlights the importance of monitoring the dissemination of such microorganisms across our continent, especially considering the increased usage of ceftazidime-avibactam in the region.

## 4. Materials and Methods

### 4.1. Strains, Identification, and Antibiotic Susceptibility Testing

Between August 2021 and July 2022, 43 ceftazidime-resistant *P. aeruginosa* isolates were isolated from 23 patients admitted to the ICU of the University Hospital of Montevideo, Uruguay. The isolates were obtained from various clinical sources, including respiratory secretions, blood culture, catheter tips, bronchoalveolar lavage, urine culture, and surgical wounds. 

For this report, we focused on *P. aeruginosa* isolates associated with infections, as determined by the infectious diseases team. We included one isolate per patient for a comprehensive microbiological characterization, and those associated with colonization were not considered.

Bacterial identification was performed using matrix-assisted laser desorption ionization-time-of-flight (MALDI-TOF) mass spectrometry (VITEK MS, bioMérieux, Marcy-l’Étoile, France). Antimicrobial susceptibility testing was assessed using the VITEK 2 system (bioMérieux, Marcy l’Étoile, France). Additionally, susceptibility to cefiderocol (FDC) was studied via disk diffusion and the minimum inhibitory concentration (MIC) to both ceftolozane/tazobactam (C/T) and ceftazidime/avibactam (CZA) were determined via E-tests (bioMérieux, Marcy l’Étoile, France) according to the manufacturer’s indications. Results were interpreted based on the Clinical and Laboratory Standards Institute (CLSI) 2022 guidelines. In accordance, when results fell within the intermediate category, they were considered as not susceptible together with the resistant ones [37]. 

### 4.2. Resistance Mechanisms Detection

Phenotypic ESBL production was assessed using a double-disk synergy test, using discs of amoxicillin-clavulanic acid and ceftazidime [38]. After analysis of the whole genome sequencing results (see below), and given the epidemiological background of *P. aeruginosa* isolates from the same setting, genes coding for *bla*_PER-1_ and *qnrVC* were searched with PCR using specific primers as previously described [39,40] and confirmed using Sanger sequencing (Unidad de Secuenciación, Hospital de Clínicas). 

### 4.3. Pulse-Field Gel Electrophoresis 

Pulse-field gel electrophoresis (PFGE) was conducted in accordance with the standard procedures recommended by PulseNet for *Escherichia coli*, *Salmonella*, and *Shigella* (PNL05) [41], with some modifications. Briefly, the isolates were cultured overnight on TSA and colonies were utilized to adjust to an 8.4 McFarland suspension in TE buffer (0.1 M Tris, 0.1 M EDTA, pH8, Sigma-Aldrich, Darmstadt, Germany). Plugs were assembled in molds by mixing 0.15 mL of the cell suspension, 0.15 mL of 1.5% low-melting point agarose (prepared in 1% SDS, TE buffer), and 0.5 g/L of proteinase K. Once solidified, plugs were placed in 2 mL of cell lysis buffer (50 mM Tris, 50 mM EDTA, 1% N-lauryl sarcosine, Sigma-Aldrich, Darmstadt, Germany) with 0.25 g/L proteinase K and incubated for 18 h at 56 °C with 150 rpm shaking in a water bath. Plugs were washed every 15 min, two times with molecular-grade water and three times with TE buffer. A 2 mm slice of each plug was cut, placed in a restriction solution containing 15 U enzyme *Spe*I and 1× buffer (ThermoFisher Scientific, Waltham, MA, USA), and incubated for 18 h at 37 °C. The slices were embedded in a 1% low-melting point agarose gel in 0.5× TBE buffer (45 mM Tris, 45 mM Boric acid, 1 mM EDTA, pH 8). *Salmonella* Braenderup H9812 digested with *Xba*I (ThermoFisher Scientific, Waltham, MA, USA) was used as a standard, as recommended by PulseNet. 

PFGE was performed in a CHEF-DR III (Bio-Rad Laboratories, Inc., Life Sciences Group, Hercules, CA, USA) device, at 14 °C, 6 V, initial and final pulse times 4 and 40 s, respectively, for 20 h. Gels were stained with 0.5 g/L ethidium bromide and photographed under UV light. Restriction patterns were analyzed using BioNumerics 6.6 software (Applied Maths, 2011). Comparisons were made by calculating the Dice coefficient (optimization 1%, tolerance 2%) and dendrograms were generated using the UPGMA method (Unweighted Pair Group Method with Arithmetic Mean). Isolates showing 100% similarity were considered identical and those with ≥80% similarity were considered clonally related [42]. 

### 4.4. Susceptibility to Combination of Antibiotics

The susceptibility to either meropenem, ciprofloxacin, amikacin, or gentamicin combined with rifampicin was assessed using the checkerboard method in microtiter plates. The fractional inhibitory concentration index (FICI) was calculated as follows: FICI = (MIC_A-B_/MIC_A_) + (MIC_B-A_/MIC_B_), where MIC_A_ and MIC_B_ are the minimum inhibitory concentration for antibiotics A and B, respectively, while MIC_A-B_ and MIC_B-A_ represent the MIC to antibiotic A in the presence of antibiotic B and MIC to antibiotic B in the presence of A, respectively. A FICI value ≤0.5 was interpreted as a synergistic effect between antibiotics A and B [43].

### 4.5. Biofilm Formation and Antibiotic Susceptibility of Mature Biofilm

Biofilm formation capability for two representative strains (HCPa02 and HCPa12) was determined using the crystal violet static model previously described, with few modifications. Briefly, 1/10 aliquots of the overnight cultures in LB broth were placed in 96 flat-bottomed well polystyrene plates at a final volume of 200 μL. After 24 h incubation at 37 °C, the wells were washed with PBS and stained with 1% crystal violet (CV) for 15 min. Excess dye was removed with PBS washes, and CV was solubilized with 95% ethanol. The biofilm biomass was quantified according to the CV optical density (OD) at 590 nm [44]. Biofilm formation categories were defined according to the OD control (ODc) value, corresponding to the OD of wells without bacteria, as follows: OD ≤ Odc = no biofilm producer; Odc < OD ≤ (2 × Odc) = weak biofilm producer; (2 × Odc) < OD ≤ (4 × Odc) = moderate biofilm producer; and (4 × Odc) < OD = strong biofilm producer [45]. *P. aeruginosa* ATCC 27853 was used as a control for strong biofilm production in all assays. 

In order to further characterize the isolates, the effects of ciprofloxacin, meropenem, gentamicin, amikacin, and rifampicin on mature biofilms were determined under the same conditions described above. After three washes with PBS to remove planktonic cells, LB alone or with different antibiotic concentrations was added over the 24 h mature biofilms. After 20 h incubation at 37 °C, planktonic cells were quantified according to the determination of DO 600 nm and their viability was confirmed with colony counting; then, they were removed and the CV staining protocol was followed as described above. 

All biofilm experiments were performed in triplicate. Differences between treatments were assessed using one-way analysis of variance (ANOVA) and Bonferroni’s post-test was used to compare pairs of groups. All analyses and graphics were performed in GraphPad Prism 5.0.

### 4.6. Short- and Long-Read Genome Sequencing

Five isolates (HCPa01, HCPa02, HCPa10, HCPa12, and HCPa16) belonging to different pulse type variants were subjected to whole genome sequencing (WGS) using short-read genome sequencing, and one of them (HCPa10) was also subjected to long-read genome sequencing, as previously described [17].

Briefly, genomic DNA was extracted using the NZY microbial gDNA Isolation kit (NZYTech Genes & Enzymes, Lisbon, Portugal). Libraries were prepared with the Nextera XT DNA Library Prep kit and Nextera XT Index kit (Illumina Inc., San Diego, CA, USA). Next-generation sequencing was performed using an Illumina MiniSeq system with a MiniSeq High Output reagent kit (Illumina Inc., San Diego, CA, USA) and a 2 × 151 bp paired-end approach. Reads were assembled with SPAdes ver. 3.11.

For the long-read genome sequencing with Oxford Nanopore Technologies, DNA libraries were prepared using a rapid sequencing kit (SQK-RAD004), loaded onto R9.4.1 flow cells (FLOMIN106) and sequenced for 8 h on a MinION device (Oxford Nanopore Technologies, Oxford, UK). Basecalling and data quality determination were assessed as previously described [17]. Genome hybrid assembly, using short and long reads, was performed with Unicycler ver. 0.4.8 [46].

### 4.7. Sequence Analysis 

The prediction of antibiotic resistance genes was performed using AMRFinderPlus v.3.11.18 [47] and ABRicate v.1.01 (https://github.com/tseemann/abricate/, accessed on 1 November 2023) with the ResFinder database. Additionally, ABRicate was used to predict virulence-coding genes using the Virulence Factors Database (VFDB). Sequence type and serotype were determined using MLST 2.0 and PAst, respectively, both available at the Center for Genomics Epidemiology site (https://cge.cbs.dtu.dk/, last accessed on 1 November 2023). 

The complete genome of HCPa10 was annotated using the RAST 2.0 suite (Rapid Annotation using Subsystem Technology) [48] and manually curated with Artemis software [49]. Comparisons with publicly available sequences were performed using BLAST (http://blast.ncbi.nlm.nih.gov/, last accessed on 1 November 2023), and physical maps were generated with EasyFig 2.1 using BLAST 2.2.18 (http://mjsull.github.io/Easyfig/, last accessed on 1 November 2023).

Genome data were deposited in the GenBank database under BioProject acc. no. PRJNA1036250, and the assembled HCPa10 chromosome under acc. no. CP139424. The new transposon number was assigned by the Transposon Registry repository [50]. 

## Figures and Tables

**Table 1:** Microbiological characteristics of one representative isolate from each patient (n = 19).

Patient ID/Strain ^‡^	Date of 1st Isolate (dd/mm/yyyy)	Sample/s (in Chronological Order)	Genes (PCR)	Minimum Inhibitory Concentration (mg/L)								CZA (E-Test)	C/T (E-Test)	FDC (DD)
				PTZ	CAZ	FEP	IPM	MEM	GM	AK	CIP			
HCPa01	11/8/2021	Blood culture, catheter tip	*bla*_PER-1_/*qnrVC*	32 (I)	≥64 (R)	≥32 (R)	≥16 (R)	8 (R)	4 (S)	≥64 (R)	0.25 (S)	16 (R)	>256 (R)	S
HCPa02	27/8/2021	Respiratory secretions, blood culture, bronchoalveolar lavage	*bla*_PER-1_/*qnrVC*	32 (I)	≥64 (R)	≥32 (R)	8 (R)	≤0.25 (S)	4 (S)	≥64 (R)	0.5 (S)	24 (R)	>256 (R)	S
HCPa03	29/8/2021	Respiratory secretions, blood culture	*bla*_PER-1_/*qnrVC*	32 (I)	≥64 (R)	≥32 (R)	8 (R)	1 (S)	4 (S)	≥64 (R)	1 (R)	24 (R)	>256 (R)	S
HCPa04	30/8/2021	Blood culture	*bla*_PER-1_/*qnrVC*	8 (S)	≥64 (R)	≥32 (R)	2 (S)	0.5 (S)	4 (S)	≥64 (R)	0.5 (S)	8 (S)	>256 (R)	S
HCPa05	3/10/2021	Respiratory secretions, blood culture, catheter tip	*bla*_PER-1_	16 (S)	≥64 (R)	≥32 (R)	≥16 (R)	0.5 (S)	4 (S)	≥64 (R)	0.25 (S)	16 (R)	>256 (R)	S
HCPa06	12/10/2021	Respiratory secretions	*bla*_PER-1_/*qnrVC*	16 (S)	≥64 (R)	≥32 (R)	1 (S)	≤0.25 (S)	4 (S)	≥64 (R)	0.5 (S)	16 (R)	>256 (R)	S
HCPa07	20/10/2021	Respiratory secretions	*bla*_PER-1_/*qnrVC*	8 (S)	≥64 (R)	≥32 (R)	2 (S)	1 (S)	8 (I)	≥64 (R)	0.25 (S)	8 (S)	128 (R)	S
HCPa08	10/12/2021	Respiratory secretions	*bla*_PER-1_	16 (S)	≥64 (R)	≥32 (R)	2 (S)	0.5 (S)	4 (S)	≥64 (R)	0.25 (S)	8 (S)	>256 (R)	S
HCPa09	9/1/2022	Respiratory secretions	*bla*_PER-1_	32 (I)	≥64 (R)	≥32 (R)	2 (S)	≤0.25 (S)	4 (S)	≥64 (R)	1 (R)	16 (R)	>256 (R)	S
HCPa10	11/1/2022	Blood culture, respiratory secretions	*bla*_PER-1_/*qnrVC*	16 (S)	≥64 (R)	≥32 (R)	2 (S)	0.5 (S)	8 (I)	≥64 (R)	0.25 (S)	16 (R)	>256 (R)	S
HCPa11	21/1/2022	Respiratory secretions	*bla*_PER-1_	16 (S)	≥64 (R)	≥32 (R)	2 (S)	0.5 (S)	4 (S)	≥64 (R)	0.25 (S)	16 (R)	128 (R)	S
HCPa12	21/1/2022	Urine, blood culture, respiratory secretions	*bla*_PER-1_	16 (S)	≥64 (R)	≥32 (R)	2 (S)	2 (S)	4 (S)	≥64 (R)	≤0.06 (S)	16 (R)	>256 (R)	S
HCPa13	24/1/2022	Surgical wound	*bla*_PER-1_	16 (S)	≥64 (R)	≥32 (R)	2 (S)	≤0.25 (S)	4 (S)	≥64 (R)	≤0.06 (S)	32 (R)	>256 (R)	S
HCPa14	12/2/2022	Respiratory secretions	*bla*_PER-1_	32 (I)	≥64 (R)	≥32 (R)	1 (S)	≤0.25 (S)	≥16 (R)	≥64 (R)	≤0.06 (S)	16 (R)	>256 (R)	S
HCPa15	13/3/2022	Respiratory secretions, blood culture	*bla*_PER-1_/*qnrVC*	16 (S)	≥64 (R)	≥32 (R)	2 (S)	1 (S)	4 (S)	≥64 (R)	0.25 (S)	8 (S)	>256 (R)	S
HCPa16	17/3/2022	Bronchoalveolar lavage	*bla*_PER-1_/*qnrVC*	16 (S)	≥64 (R)	≥32 (R)	2 (S)	≤0.25 (S)	4 (S)	≥64 (R)	0.25 (S)	16 (R)	>256 (R)	S
HCPa17	27/3/2022	Respiratory secretions, surgical wound, bronchoalveolar lavage, urine	*bla*_PER-1_	16 (S)	≥64 (R)	≥32 (R)	2 (S)	1 (S)	4 (S)	≥64 (R)	0.25 (S)	16 (R)	>256 (R)	S
HCPa18	7/4/2022	Respiratory secretions	*bla*_PER-1_/*qnrVC*	16 (S)	≥64 (R)	≥32 (R)	2 (S)	≤0.25 (S)	8 (I)	≥64 (R)	0.125 (S)	32 (R)	>256 (R)	S
HCPa19	17/7/2022	Respiratory secretions, catheter tip	*bla*_PER-1_/*qnrVC*	16 (S)	≥64 (R)	≥32 (R)	2 (S)	≤0.25 (S)	4 (S)	≥64 (R)	≥4 (R)	8 (S)	48 (R)	S

**^‡^** Underlined strains were selected for whole genome sequencing. Abbreviations: PTZ, piperacillin-tazobactam; CAZ, ceftazidime; FEP, cefepime; IPM, imipenem; MEM, meropenem; GM, gentamicin; AK, amikacin; CIP, ciprofloxacin; CZA, ceftazidime-avibactam; C/T, ceftolozane-tazobactam; FDC, cefiderocol; DD, disk diffusion method.

**Table 2:** Checkerboard assay results of the combined effect of rifampicin with either meropenem, gentamicin, amikacin, or ciprofloxacin for strains HCPa01 and HCPa12.

MIC ^†^	HCPa01	HCPa12		MIC	HCPa01	HCPa12	MIC	HCPa01	HCPa12		MIC	HCPa01	HCPa12
RD_S_	16	16		RD_S_	16	16	RD_S_	16	16		RD_S_	16	16
MEM_S_	8	2		GM_S_	4	4	AK_S_	64	64		CIP_S_	0.25	0.06
RD_C_	4	2	4	RD_C_	4	4	RD_C_	4	4	0.5	RD_C_	8	8
MEM_C_	2	0.5	0.06	GM_C_	0.5	0.5	AK_C_	8	8	16	CIP_C_	0.06	0.03
FICI ^§^	0.5	0.375	0.28	FICI	0.375	0.375	FICI	0.375	0.375	0.28	FICI	0.74	1

**^†^** MIC, Minimum inhibitory concentration expressed in mg/L for each single antibiotic (subfix S) and in combination (subfix C). **^§^** Fractional inhibitory concentration index. Antibiotic abbreviations: RD, rifampicin; MEM, meropenem; GM, gentamicin; AK, amikacin; CIP, ciprofloxacin.

## Data Availability

Whole genome sequencing data were deposited in GenBank database under BioProject acc. no. PRJNA1036250.
